# Comparative studies of urolithins and their phase II metabolites on macrophage and neutrophil functions

**DOI:** 10.1007/s00394-020-02386-y

**Published:** 2020-09-22

**Authors:** Aneta Bobowska, Sebastian Granica, Agnieszka Filipek, Matthias F. Melzig, Thomas Moeslinger, Jürgen Zentek, Aleksandra Kruk, Jakub P. Piwowarski

**Affiliations:** 1 Department of Pharmacognosy and Molecular Basis of Phytotherapy, Faculty of Pharmacy, Medical University of Warsaw, Banacha 1, 02-097 Warsaw, Poland; 2 Department of Pharmaceutical Biology, Institute of Pharmacy, Freie Universität Berlin, Berlin, Germany; 3 Institute of Physiology, Center for Physiology and Pharmacology, Medical University of Vienna, Vienna, Austria; 4 Institute of Animal Nutrition, Freie Universität Berlin, Berlin, Germany

**Keywords:** Ellagitannins, Inflammation, Urolithins, Phase II metabolism, Postbiotics

## Abstract

**Purpose:**

Ellagitannins are high molecular weight polyphenols present in high quantities in various food products. They are metabolized by human and animal gut microbiota to postbiotic metabolites-urolithins, bioavailable molecules of a low molecular weight. Following absorption in the gut, urolithins rapidly undergo phase II metabolism. Thus, to fully evaluate the mechanisms of their biological activity, the in vitro studies should be conducted for their phase II conjugates, mainly glucuronides. The aim of the study was to comparatively determine the influence of urolithin A, iso-urolithin A, and urolithin B together with their respective glucuronides on processes associated with the inflammatory response.

**Methods:**

The urolithins obtained by chemical synthesis or isolation from microbiota cultures were tested with their respective glucuronides isolated from human urine towards modulation of inflammatory response in THP-1-derived macrophages, RAW 264.7 macrophages, PBMCs-derived macrophages, and primary neutrophils.

**Results:**

Urolithin A was confirmed to be the most active metabolite in terms of LPS-induced inflammatory response inhibition (TNF-α attenuation, IL-10 induction). The observed strong induction of ERK1/2 phosphorylation has been postulated as the mechanism of its action. None of the tested glucuronide conjugates was active in terms of pro-inflammatory TNF-α inhibition and anti-inflammatory IL-10 and TGF-β1 induction.

**Conclusion:**

Comparative studies of the most abundant urolithins and their phase II conjugates conducted on human and murine immune cells unambiguously confirmed urolithin A to be the most active metabolite in terms of inhibition of the inflammatory response. Phase II metabolism was shown to result in the loss of urolithins’ pharmacological properties.

**Electronic supplementary material:**

The online version of this article (10.1007/s00394-020-02386-y) contains supplementary material, which is available to authorized users.

## Introduction

Ellagitannins are high-molecular-weight polyphenols present in high quantities in various food products and medicinal plants. Different health benefits and biological activities are attributed to these natural products being based on the dietary and clinical prospective and retrospective studies [1]. The impaired health conditions in which ellagitannin-containing products were shown to express preventive or curative effects are in particular diseases with the inflammatory background such as cardiovascular diseases and intestinal inflammations [2–5]. Despite the effectiveness of these products has been unambiguously confirmed in clinical and epidemiological studies, due to the low bioavailability of ellagitannins, this group of compounds cannot be directly considered as factors, which trigger the biological effects at the systemic level.

New light on the mechanisms standing behind the health benefits of ellagitannins has been put by the studies on their metabolism by human gut microbiota, which was shown to metabolize ellagitannins of different structures and origin to the series of low-molecular-weight postbiotic metabolites-urolithins [6]. In contrast to parental molecules, urolithins are highly lipophilic and thus can easily penetrate the biological barriers what was confirmed in human and on different animal models [7]. Studies conducted on in vitro and in vivo models have revealed that these metabolites are active especially towards inhibition of the inflammatory response. In particular, urolithin A (UA) was consistently shown to possess strong and structure-specific anti-inflammatory properties [8–16].

However, according to the pharmacokinetic studies on ellagitannin-containing products as well as pure urolithins, after absorption in the gut they rapidly undergo metabolism by phase II enzymes, what results in their presence in the serum, tissues, and the urine mainly in the form conjugated with glucuronic acid [17–21]. These observations led to the rise of controversies regarding the translation of the in vitro results for non-conjugated urolithins to their systemic activity following oral ingestion [7]. Limited number of preliminary in vitro studies have shown a loss or alterations in the activity, which were associated with phase II metabolism of urolithins [9, 22, 23].

In order to fully evaluate the biological activities of urolithins with regard to their metabolism and disposition, the in vitro studies should necessarily acknowledge their phase II metabolites, especially glucuronides as major conjugates. The low number of bioactivity studies conducted for urolithin glucuronides is caused by the lack of commercially available chemical standards. During our previous studies we have isolated urolithin glucuronides, namely iso-urolithin A 3-*O*-glucuronide (iUA) and urolithin B 3-*O*-glucuronide (GUB), and a mixture of two inseparable isomers of urolithin A: 3-*O*-glucuronide and 8-*O*-glucuronide (GUA) from a urine of a volunteer ingesting ellagitannin-rich food products [24]. Using a large-scale gut microbiota ex vivo cultures iso-urolithin A-a compound characteristic for urolithin metabotype B, was obtained [6].

The aim of the study was to comprehensively determine the influence of dominating urolithins and their respective phase II metabolites on inflammatory response of human immune cells focusing not only on their anti-inflammatory activity and its molecular mechanisms, but also on their potential contribution to the processes associated with active resolution of the inflammation. In order to achieve a broader view on the comparisons in metabolites activities, diverse in vitro models were applied including human cell line (THP-1 monocytes), murine cell line (RAW 264.7 macrophages), and primary human immune cells (PBMCs and neutrophils).

## Materials and methods

### Chemicals and reagents

Urolithins A and B (UA, UB, respectively) were synthetized according to the previously described method [25]. Iso-urolithin A (iUA) was isolated from gut microbiota cultures according to [6]. Iso-urolithin A 3-*O*-glucuronide, mixture of urolithin A 3-*O*-glucuronide and 8-*O*-glucuronide (GUA) and urolithin B 3-*O*-glucuronide were isolated from human urine according to [24]. The identity and purity of compounds were confirmed by ^1^H NMR and UHPLC-DAD-MS methods. The compounds were of > 95% purity. Phorbol myristate acetate (PMA) (Sigma-Aldrich GmbH, Steinheim, Germany); LPS from *E. coli* (Invivogen, San Diego, CA, USA); parthenolide, roscovitine, and genistein (Sigma-Aldrich, St. Louis, MO, USA). Urolithins, respective glucuronides and positive controls’ stock solutions were prepared in DMSO (Sigma-Aldrich) final concentration of DMSO in cell cultures was 0.2% and was used as stimulated and non-stimulated control throughout the conducted experiments.

### THP-1 monocyte culture and differentiation

THP-1 human monocytic cell line was purchased from DSMZ (Braunschweig, Germany). Cells were cultivated at 37 °C under humidified 5% CO_2_ in the RPMI 1640 (Biochrom, Cambridge, UK) medium containing 10% FBS (Biochrom) and 2 mM glutamine (Biochrom). In order to generate THP-1 macrophages, THP-1 cells were primed for 48 h with 12.5 ng/mL PMA followed by 24 h resting. The differentiation of the cells was confirmed by changes in morphology, phagocytosis, increased expression of differentiation markers CD11b and CD14 and their ability to respond to LPS. After 24 h, cells were incubated with urolithins and respective glucuronides at the concentration of 40 μM for 1 h prior to stimulation with LPS from *E. coli* (10 ng/mL).

### RAW 264.7 macrophages culture

The mouse monocyte/macrophage cell line RAW 264.7 (ATCC TIB 71) was cultured in Dulbecco’s Modified Eagle’s Medium (DMEM, Biochrom) supplemented with 10% FBS (Biochrom), 25 mM HEPES (Biochrom), 50 U penicillin/mL and 50 µg streptomycin/mL at 37 °C, 5% CO_2_, and 95% humidity. Cells were studied between passages 7 and 30. LPS was used at the concentration of 10 ng/mL for macrophage stimulation.

### Isolation and cultivation of monocyte/macrophage cells

Immediately after collection, 9 mL heparinised blood was subjected to a twofold dilution with PBS. Next, the blood was layered over 9 mL Ficoll Hypaque density gradient (LSM 1077, PAA, Laboratories GmbH, Austria) and centrifuged (1800 rpm, 20 min, 4 °C). The mononuclear cell band was removed by the aspiration, washed with cold PBS (PAA,), and centrifuged. The cells were suspended in RPMI 1640 medium with l-glutamine and HEPES (PAA), antibiotics: penicillin (100 U/mL), streptomycin (100 μg/mL), amphotericin and gentamycin (2.5 μg/mL) (Sigma-Aldrich), and autologous serum (20%). To allow the adherence of monocytes/macrophages, the peripheral blood mononuclear cells suspension was placed in 12-well tissue culture plates (2 × 10^6^ per well) and incubated for 2 h at 37 °C under humidified 5% CO_2_ air. After this time, non-adherent cells were removed and washed twice with PBS, whereas adherent cells were cultivated in the same medium and conditions for next 7 days. The medium and autologous serum were replaced every 2 days [26].

### Human primary neutrophils isolation

The buffy coat was prepared from peripheral venous blood collected from healthy human donors (20–35 years old) at the Warsaw Blood Donation Center. All Donors declared that they were non-smokers and did not take any medications. They were confirmed to be healthy and all tests carried out showed values within a normal range. For each experiment buffy coats obtained from three donors were used. Neutrophils were isolated using a standard method by dextran sedimentation and centrifugation in a Ficoll Hypaque gradient [27]. The purity of neutrophil preparation was over 97%. After isolation, cells were suspended in HBSS (PAA), PBS (PAA) or culture medium (RPMI 1640) and maintained at 4 °C before use.

### Cell viability

Cytotoxicity of urolithins and respective glucuronides was determined using a standard MTT test. After 24 h of incubation of differentiated THP-1 cells with compounds at the concentration of 40 μM with or without stimulation with LPS (PAA), cells were washed twice with fresh culture medium and afterwards 300 μL of the medium containing MTT (Carl Roth GmbH, Karlsruhe, Germany) at the concentration of 0.5 mg/mL was added. After 1 h of incubation in 37 °C, the medium was removed, cells were washed with fresh medium, and formazan crystals were dissolved in 300 μL of DMSO (Sigma-Aldrich). Absorbance was measured at 580 nm.

### Phagocytosis assay

Differentiated THP-1 macrophages were treated with compounds (40 µM) for 1 h. The phagocytosis was determined using Phagocytosis Assay Kit (IgG FITC) (Cayman Chemicals, Ann Arbor, MI, USA). After the treatment with compounds, 20 µL of FITC labelled beads suspension was added to the culture and the cells were incubated at 37 °C for 4 h. The level of phagocytosis was determined using flow cytometry (Cytoflex; Beckman Coulter). To distinguish cells which have phagocytosed the beads from those binding the beads at the surface, a trypan blue quenching solution was added before the measurement.

### Enzyme-linked immunosorbent assay (ELISA)

After the stimulation of THP-1 macrophages with LPS, the culture media were collected and the levels of TNF-α and IL-10 were measured using ELISA kits (BD Biosciences, Franklin Lakes, NJ, USA) according to the manufacturer’s protocols. Results were represented as fold changes relative to the control.

### RNA extraction and reverse transcription

After 24-h incubation of THP-1 macrophages with LPS, the cell culture media was removed, and the wells were washed two times with cold PBS. Cells were scratched to 500 μL of RNALater, which was added to each well. The total cellular RNA was extracted using NucleoSpin RNA Clean-up (Macherey–Nagel, Düren, Germany) according to the manufacturer’s instructions. RNA samples were treated with PureLink DNase (Invitrogen, Carlsbad, CA, USA). The quality and quantity of isolated RNA was evaluated using 2100 Bioanalyzer (Agilent Technologies, Palo Alto, CA, USA). Total RNA was reversely transcribed to cDNA using Superscript III reverse Transcriptase kit (Life Technologies, Carlsbad, CA, USA) according to the manufacturer’s protocol.

### Real-time PCR analysis

The levels of mRNA expression for TNF-α and TGF-β1 in THP-1 macrophages were quantified by Brilliant II SYBR Green QPCR Kit (Agilent Technologies, Palo Alto, CA, USA) using Aria Mx Real-Time PCR system (Agilent Technologies). For the qPCR experiments primers against the sequences of human TNF-α (sense: 5′-GGCCTCTGTGCCTTCTTTTG-3′, antisense: 5′-CCTCAGCAATGAGTGACAGT-3′), TGF-β1 (sense: 5′-CGTGGAGCTGTACCAGAAATA-3′, antisense: 5′-TCCGGTGACATCAAAAGATAA-3′), and the housekeeping gene β-actin (ACTB) (sense: 5′-TTGCCGACAGGATGCAGAAGGA-3′, antisense: 5′-AGGTGGACAGCGAGGCCAGGAT-3′) were purchased from RealTimePrimers (Elkins Park, USA). The cycling conditions were 95 °C for 10 min followed by 40 cycles of denaturing at 95 °C for 15 s and annealing/extension for 1 min at 60 °C. Relative gene expression was calculated with the 2^−ΔΔCt^ method, and ACTB was chosen as the reference gene. Results were represented as fold expression change relative to the control group.

### Protein isolation

After 35 min of stimulation THP-1 macrophages with LPS, the medium was aspirated, and the cells were washed with ice-cold PBS twice. Afterwards, the cells were lysed with cOmplete™ Lysis-M (Roche, Basel, Switzerland) containing Protease and Phosphatase inhibitor cocktail (Roche, Basel, Switzerland) and centrifuged at 14,000*g* for 15 min at 4 °C. For NFκB p65 nuclear translocation after LPS stimulation the cells were lysed using Mammalian Nuclear and Cytoplasmic Protein Extraction Kit (SERVA, Heidelberg, Germany). The total protein concentration of the cell and nuclear lysates was quantified with BCA protein assay kit (Bio-Rad, Hercules, CA, USA) using BSA as a standard. After equilibration of protein concentration between the samples, the lysates were boiled with 4xLaemmli Sample Buffer (Bio-Rad) and stored in − 20 °C.

### Western blot analysis

The protein lysates were separated by 10 or 12.5% SDS-PAGE and transferred to nitrocellulose membrane Amersham Protran (Sigma-Aldrich). The membrane was blocked with 5% skimmed milk powder (Sigma-Aldrich) or 5% BSA (Sigma-Aldrich) in Tris-buffered saline (Roth GmbH, Karlsruhe, Germany) containing 0.1% (v/v) Tween 20 (SERVA), then incubated overnight at 4 °C with primary rabbit monoclonal antibodies: anti-NFκB p65 (Abcam, Cambridge, UK), anti-TAK1 (Abcam), anti-p-TAK1, Thr184/187 (Cell Signaling Technology, USA), anti-IκBα (Abcam), anti-p-IκBα Ser32 (Cell Signaling Technology, USA), anti-p38 MAPK (Cell Signaling Technology), anti-p-p38 MAPK, Thr180/Tyr182 (Cell Signaling Technology), anti-SAPK/JNK (Cell Signaling Technology), anti-p-SAPK/JNK, Thr183/Tyr185 (Cell Signaling Technology), anti-ERK1/2 (Cell Signaling Technology), anti-p-ERK1/2, Thr202/Tyr204 (Cell Signaling Technology), and anti-beta Actin, polyclonal (Abcam). The membranes were washed three times and incubated with the secondary antibody at a dilution of 1:3000 (v/v). HRP-conjugated goat anti-rabbit IgG antibody (Abcam) was used as a secondary antibody. The protein bands were detected with SignalFire ECL Reagent (Cell Signaling Technology) and visualized using PXi 4 Touch (SYNGENE, Frederick, MD, USA). Beta-actin was used as a loading control. The protein level was determined by normalization to that of beta-actin. Relative expression was determined as the ratio of the intensity of the protein band to that of the corresponding control band and finally represented as fold increase over the control group.

### FACS analysis of IL-10 receptor expression

The expression of IL-10 receptor on the surfaces of monocytes/macrophage cells was determined by flow cytometry using FACSCalibur (BD Pharmingen, San Diego, CA, USA). Cells were incubated with LPS at a concentration of 100 ng/mL for 1 h, and they were then incubated with each compound for 24 h. All solutions were added to the cells on the second day of incubation. Cells then were removed, centrifuged (13,000 RPM, 4 °C, 1 min), suspended in PBS (100 μL), and incubated with the antibody (PE Rat Anti-human IL-10, BD Pharmingen, BD Bioscience, USA) for 20 min at 4 °C. The mean fluorescence intensity in the gated cell population was measured (10,000 cells per sample) and analyzed by flow cytometry. The results were expressed as the percent of cells expressing IL-10 receptor in comparison to control cells stimulated by LPS.

### Nitrite analysis

Nitrite in RAW 264.7 cell culture supernatants was determined spectrophotometrically using the Griess reagent (0.5% sulfanilic acid, 0.002% *N*-1-naphtyl-ethylenediamine dihydrochloride, 14% glacial acetic acid). Absorbance was measured at 550 nm with baseline correction at 650 nm and nitrite concentration was determined using sodium nitrite as a standard.

### Western blotting for inducible nitric oxide synthase

RAW 264.7 cells were lysed in ice-cold buffer containing 25 mM monosodium phosphate (pH = 7.4), 75 mM NaCl, 5 mM EDTA, 1% Triton X-100, and centrifuged at 20,000*g* for 15 min at 4 °C. The cytosolic proteins (12 μg per lane) were separated by 12% SDS–polyacrylamide gel electrophoresis. Proteins were transferred to nitrocellulose filters and then immunoblotted with a rabbit anti-iNOS (obtained from Biomol, Hamburg, Germany) or a rabbit anti-actin polyclonal antibody at a 1:2000 dilution. Anti-rabbit alkaline phosphatase-conjugated antibody was used as a secondary antibody at a dilution of 1:7500. Finally, the blots were incubated with 5-bromo-4-chloro-3-indolyl-phosphate/nitro blue tetrazolium reagent (Promega) for 10–15 min.

### Preparation of nuclear extracts

RAW 264.7 cells (8 × 10^5^) were seeded in 6-well plates. LPS and indicated amounts of urolithins were added simultaneously to the culture medium for 25 min. Cells were washed twice, resuspended in 500 μL lysis buffer (10 mM Tris–HCl (pH = 7.5), 2 mM MgCl_2_, 10 mM KCl, 0.5 mM dithiothreitol, and 0.6% IGEPAL CA-630), and vortexed for 30 s. Nuclei were pelleted by centrifugation at 10 000 g for 2 min at 4 °C, resuspended in 30 μL of a buffer containing 20 mM Tris–HCl (pH = 7.8), 5 mM MgCl_2_, 420 mM NaCl, 0.2 mM EGTA, 0.5 mM dithiothreitol, and 25% (v/v) glycerol, vortexed for 30 s, and incubated on ice for 15 min. Lysates were centrifuged at 20,000*g* for 5 min at 4 °C. The supernatants were kept as aliquots at – 70 °C until analyzed by Western blotting as described above. Rabbit anti-NF-κB (p65) primary antibodies (Santa Cruz Biotechnology, Santa Cruz, CA, USA) were diluted for optimal signal.

### Neutrophil apoptosis

Neutrophils’ viability and apoptosis were determined by staining with propidium iodide (PI) and Annexin V-FITC using Annexin V Apoptosis Detection Kit I (BD Pharmingen, San Diego, CA, USA) following the manufacturer’s instructions. Neutrophils (2 × 10^6^ per mL) were cultured in a 24-well plate in RPMI 1640 medium (as above) in the absence or presence investigated compounds at a final concentration 40 µM added 1 h before the stimulation with LPS (100 ng/mL). Cells were collected, centrifuged (2000 rpm; 10 min; 4 °C), and resuspended in 100 µl of binding buffer. Next 5 μL of Annexin and 5 μL of propidium iodide solutions were added. The mixture was vortexed and incubated for 15 min at room temperature in the darkness. Then, 400 µl of cold binding buffer was added and the cells were analyzed by flow cytometry (FACSCalibur) within 1 h after labeling and data from 10,000 events were recorded. Before each experiment compensation (using untreated cells, cells with Annexin V and cells with iodide propidium) was performed and quadrants have been set. Roscovitine at a concentration of 40 µM was used as a positive control.

### Impact on β-glucuronidase release (azurophilic granules)

After isolation, neutrophils were resuspended in HBSS (6 × 10^5^ cells/mL) with urolithins and respective glucuronides at the concentration of 40 μM, primed with cytochalasin B (10 μM) for 5 min and then stimulated with f-MLP (1 μM) for 10 min. The neutrophils were centrifuged (2000 rpm; 10 min; 4 °C). The amount of released β-glucuronidase in supernatants was determined using a human β-glucuronidase ELISA Kit (Wuhan Fine Biological Technology Co., Ltd, Wuhan, China) according to the manufacturer’s instructions.

### Impact on specific granules release

After isolation, neutrophils were resuspended in HBSS (6 × 10^5^ cells/mL) with urolithins and respective glucuronides at the concentration of 40 μM, primed with cytochalasin B (10 μM) for 5 min and then stimulated with f-MLP (1 μM) for 10 min. The neutrophils were centrifuged (12,000*g*; 15 s; 4 °C), washed once with cold HBSS and stained with anti-CD66b-FITC antibody (BD Pharmingen, San Diego, CA, USA), incubated 30 min. in darkness, centrifuged (12,000*g*; 15 s; 4 °C), washed with HBSS with 0.01% BSA, centrifuged, resuspended in HBSS with 0.01% BSA and analyzed using flow cytometry (FACSCalibur) within 0.5 h after labelling, and data from 10,000 events were recorded. Genistein at the concentration of 40 μM was used as a positive control.

### Statistical analysis

Results of at least three independent experiments performed in triplicate are shown as mean values ± SD. Statistical significance of differences between means was determined by one-way ANOVA. For comparison of results with the control group, Dunnett’s post hoc test was used. To compare the differences between the inhibitory activities of compounds, Tukey’s post hoc test was performed. Results with *p* value < 0.05 were considered statistically significant. All analyses were performed using Statistica 13 software.

## Results

None of the tested compounds exhibited cytotoxic effect on THP-1 macrophages (MTT test, Fig. S1) and on human primary neutrophils (propidium iodide staining followed by FACS analysis, Fig. S2).

**Fig. 1:**
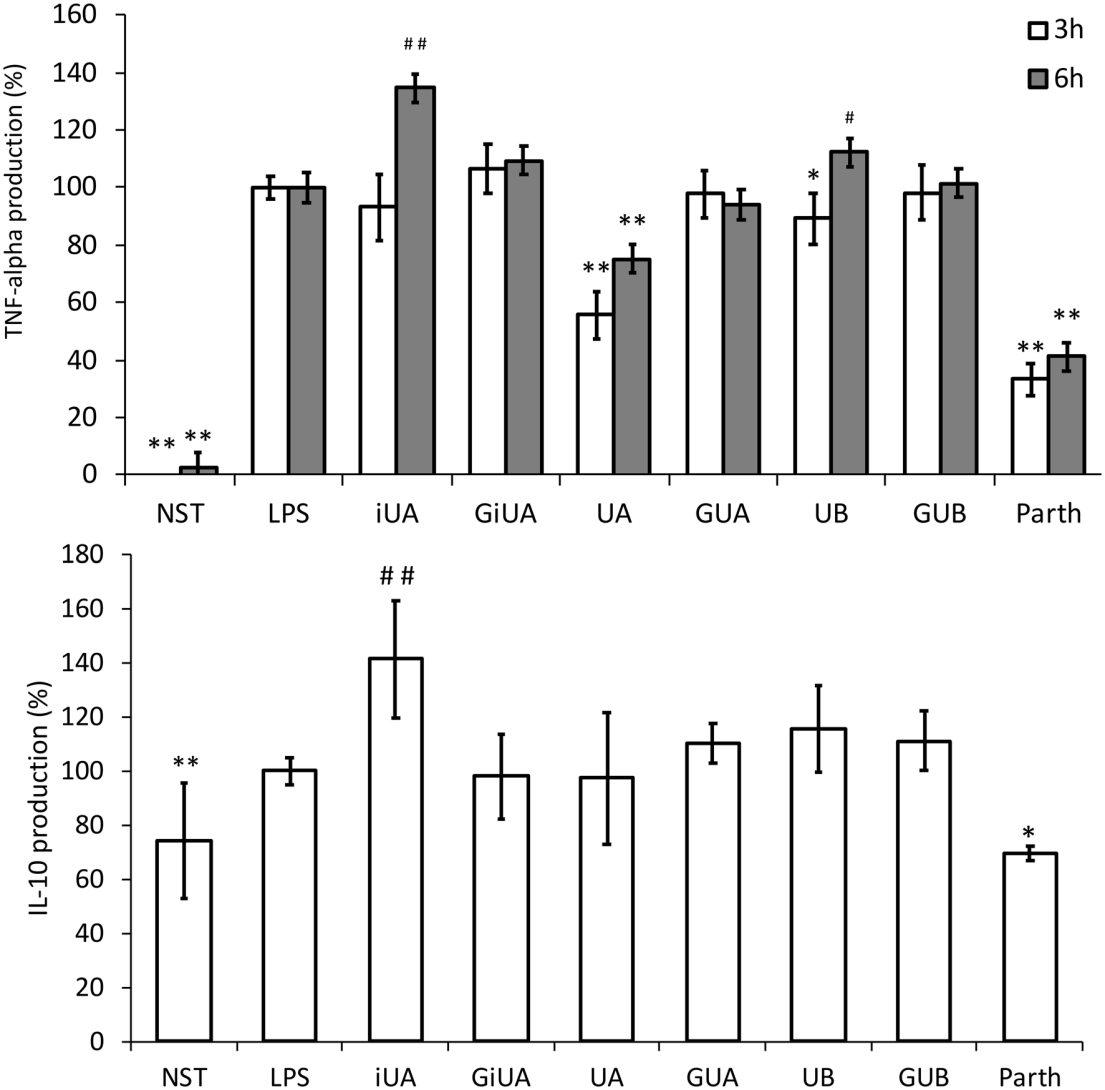
Effects of tested urolithins and respective glucuronides at the concentration of 40 μM on LPS-induced TNF-α and IL-10 production. PMA-differentiated THP-1 cells were preincubated for 1 h with iso-urolithin A, urolithin A and B (iUA, UA, UB) and their respective glucuronides (GiUA, GUA, GUB) at the concentration of 40 μM and stimulated with LPS (10 ng/mL). Statistical significance: *p < 0.05, **p < 0.001 decrease versus stimulated control (Dunnettʼs post hoc test); ^#^*p* < 0.05, ^##^*p* < 0.001 increase versus stimulated control; LPS—stimulated control; NST—non-stimulated control. Parth-parthenolide at the concentration of 5 μM (positive control). Data are expressed as mean ± SD of three separate experiments performed in triplicate. Mean ± SD and *p* values are provided in Supplementary material Table S3

**Fig. 2:**
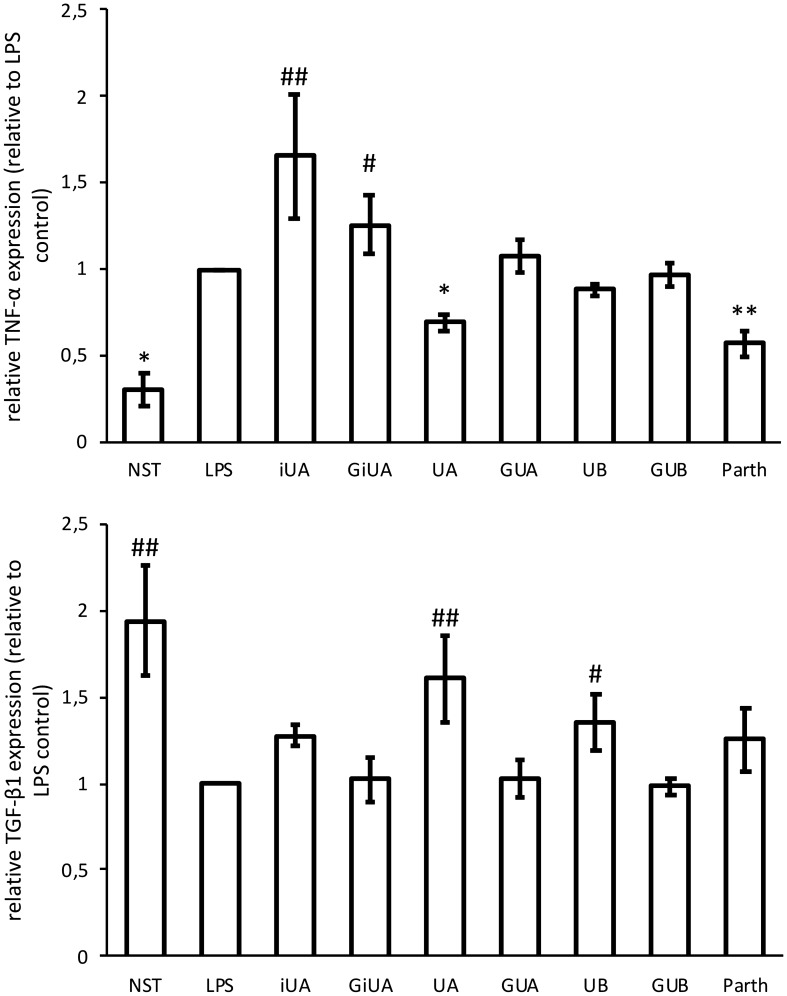
Effects of tested urolithins and respective glucuronides at the concentration of 40 μM on LPS induced TNF-α and TGF-β1 mRNA expression in THP-1 macrophages. PMA-differentiated THP-1 cells were preincubated for 1 h with iso-urolithin A, urolithin A and B (iUA, UA, UB) and their respective glucuronides (GiUA, GUA, GUB) at the concentration of 40 μM and stimulated with LPS (10 ng/mL) for 24 h. Real-time RT-PCR analysis was performed as described in Sect. “Materials and methods”. Changes in mRNA expression were normalized to β-actin. *p < 0.05, **p < 0.001 decrease versus stimulated control (Dunnettʼs post hoc test); ^#^*p* < 0.05, ^#^*p* < 0.001—statistically significant increase versus stimulated control; LPS—stimulated control; NST—non-stimulated control. Parth-parthenolide at the concentration of 5 μM (positive control). Data are expressed as mean ± SD of three separate experiments performed in duplicate. Mean ± SD and *p* values are provided in Supplementary material Table S4

**Fig. 3:**
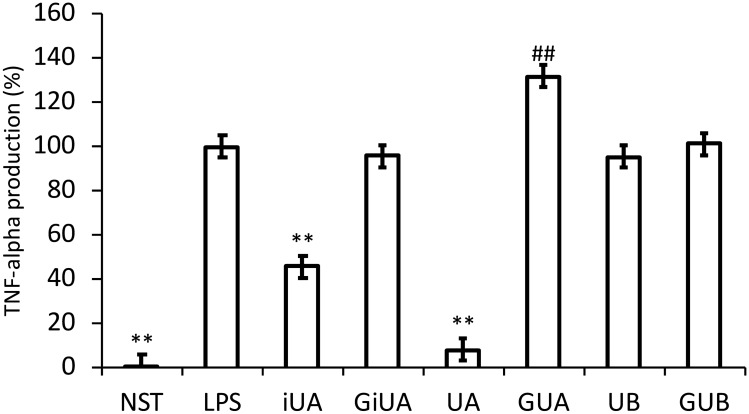
Effects of tested urolithins and respective glucuronides at the concentration of 40 μM on LPS-induced TNF-α. PBMCs were incubated with iso-urolithin A, urolithin A and B (iUA, UA, UB) and their respective glucuronides (GiUA, GUA, GUB) at the concentration of 40 μM and stimulated with LPS (100 ng/mL). Statistical significance: *p < 0.05, **p < 0.001 decrease versus stimulated control (Dunnettʼs post hoc test); ^#^*p* < 0.05, ^#^*p* < 0.001—statistically significant increase versus stimulated control; LPS—stimulated control; NST—non-stimulated control. Data are expressed as mean ± SD of three separate experiments performed in triplicate. Mean ± SD and *p* values are provided in Supplementary material Table S5

The first and most abundant cytokine produced by macrophages following inflammatory stimuli is TNF-α. Strong inhibition of TNF-α production was observed for UA, 3 and 6 h after LPS stimulation (by 44.3 ± 8.1% and 24.8 ± 7.7% respectively). In contrast, its isomer-iUA after 6 h caused significant increase in TNF-α level by 34.6 ± 9.8% (Fig. 1). The incubation of cells with respective glucuronide conjugates GUA and GiUA did not result in any changes in this cytokine production. The qPCR determination of mRNA has shown that the decrease in the TNF-α protein levels were strictly dependent on the modification of the expression of mRNA for TNF-α (Fig. 2). The inhibition of TNF-α production was compared with positive control-parthenolide (inhibitor of NFκB pathway), which at the concentration of 5 μM decreased the level of TNF-α by 66.8 ± 5.5% after 3 h and by 58.9 ± 8.8% after 6 h (Figs. 1, 2). The inhibitory activity of UA towards TNF-α production was additionally confirmed on human primary macrophages. UA at the concentration of 40 μM showed much stronger inhibition (91.7 ± 5.5%) than on THP-1 macrophages. In contrast to the observations conducted on THP-1 macrophages, where iUA induced TNF-α production, in primary cells it inhibited the production of this cytokine by 54.4 ± 3.4% (Fig. 3). Similarly, as on THP-1 cell line model, respective glucuronides remained inactive towards suppression of the inflammatory response of primary macrophages.

**Fig. 4:**
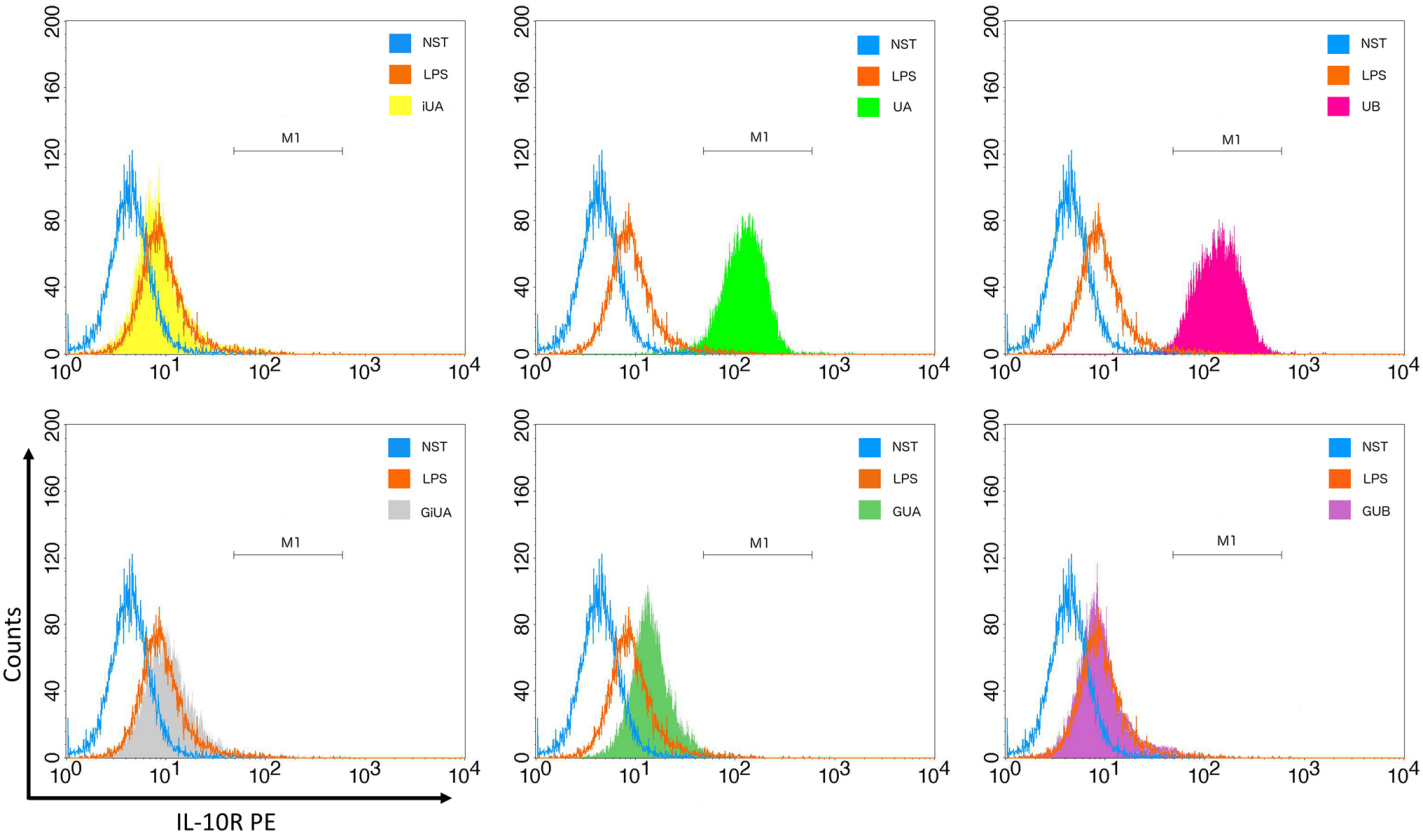
Effects of tested urolithins and respective glucuronides at the concentration of 40 μM on IL-10 receptor expression. PBMC macrophages were incubated with iso-urolithin A, urolithin A and B (iUA, UA, UB) and their respective glucuronides (GiUA, GUA, GUB) at the concentration of 40 μM and stimulated with LPS (100 ng/mL). Presented data are representative for three independent experiments performed with cells isolated from independent donors

Macrophages are known to be responsible for the resolution of inflammation by release of anti-inflammatory cytokines such as IL-10, which inhibits the proinflammatory cytokine production (such as TNF-α and IL-1β). They also secrete TGF-β1, which has a broad range of anti-inflammatory functions. TGF-β1 can induce neutrophil apoptosis, inhibit T-cell proliferation, and induce monocyte differentiation to macrophages and is a potent suppressor of the proinflammatory monocyte activation [28]. UA and UB were stimulating the expression of TGF-β1 in THP-1 macrophages by 60.0 ± 25.0% and 35.0 ± 16.7% respectively. As in previous experiments, the corresponding glucuronides remained inactive (Fig. 2). The stimulation of IL-10 production was only observed for iUA by 41.4 ± 21.6% (Fig. 1). Studies on IL-10 receptor surface expression have shown its strong increase in primary macrophages incubated with UA and UB (Fig. 4).

Another crucial step in the resolution of inflammation is the phagocytosis of neutrophils and the cellular debris by macrophages [28]. In order to evaluate the potential influence of urolithins on this process we monitored their impact on phagocytosis of FITC-labelled IgG coated beads. The phagocytosis of beads by THP-1 macrophages was shown to be time dependent (Fig. S3). In order to evaluate the changes in the phagocytosis induced by the tested compounds, the determination of their activity was conducted 4 h after incubation,; however, neither urolithin aglycones nor glucuronides were active in the tested model (Fig. S3).

**Fig. 5:**
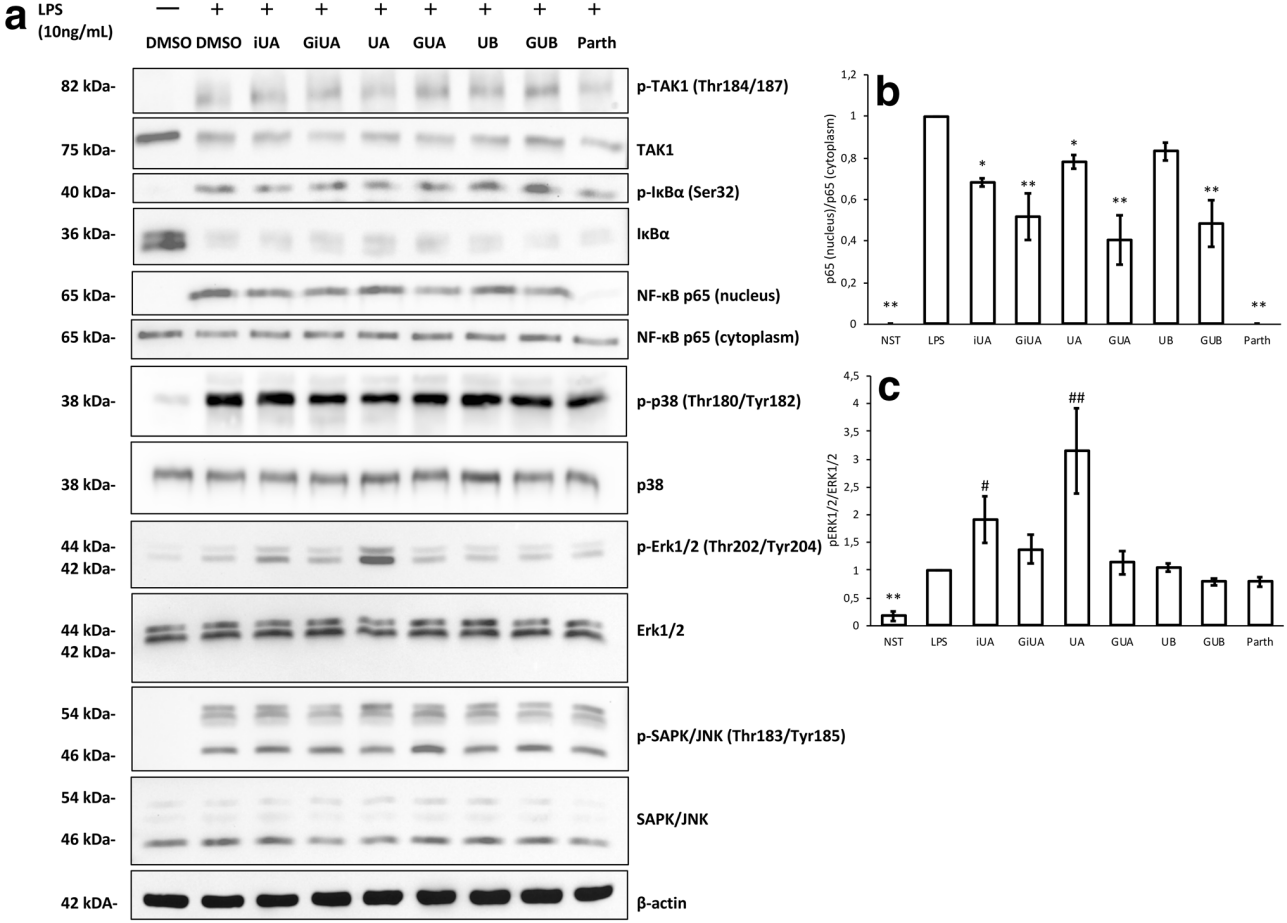
Effects of tested urolithins and respective glucuronides at the concentration of 40 μM on LPS-induced NF-κB and MAPK pathway activation in THP-1 macrophages. PMA-differentiated THP-1 cells were preincubated for 1 h with iso-urolithin A, urolithin A and B (iUA, UA, UB) and their respective glucuronides (GiUA, GUA, GUB) at the concentration of 40 μM and stimulated with LPS (10 ng/mL) for 35 min. Western blotting analysis was performed as described in “Materials and methods”. The chart B represents the changes in p65 protein levels in nuclear fractions normalized to p65 protein levels in cytosolic fractions. Chart C represents the changes in phospho-ERK1/2 normalized to total ERK1/2. Statistical significance: *p < 0.05, **p < 0.001 decrease versus stimulated control (Dunnettʼs post hoc test); ^#^*p* < 0.05, ^#^*p* < 0.001—statistically significant increase versus stimulated control; LPS—stimulated control; NST—non-stimulated control. Parth-parthenolide at the concentration of 5 μM (positive control). Densitometric analysis was performed using ImageJ software. Mean ± SD and *p* values are provided in Supplementary material Table S6. All presented data are representative for three independent experiments

The studies on the molecular mechanism of the cytokine release inhibition/stimulation have shown that all of the compounds, including urolithin glucuronides influenced the NFκB pathway and the inhibition took place between IκBα degradation and p65 nuclear translocation. The inhibitory activity was apparently much more pronounced for GUA and GUB than for respective free urolithins. The inhibition was not as strong as observed for parthenolide at the concentration of 5 μM, which has almost completely attenuated the entry of p65 to the nucleus (Fig. 5). Much stronger inhibition of NFκB p65 nuclear translocation to the nucleus observed for GiUA, GUA and GUB (Fig. 5b) was,, however, not congruent with their lack of influence on TNF-α, IL-10 and TGF-β1 production and does not explain outstanding activity of UA on TNF-α production. Thus, the influence on MAP kinases phosphorylation was examined. Neither urolithins, nor their glucuronides influenced the LPS-induced p38 and SAPK/JNK phosphorylation. Significant changes were observed in the ERK1/2 phosphorylation, which was strongly stimulated by UA and slightly by iUA (Fig. 5).

**Fig. 6:**
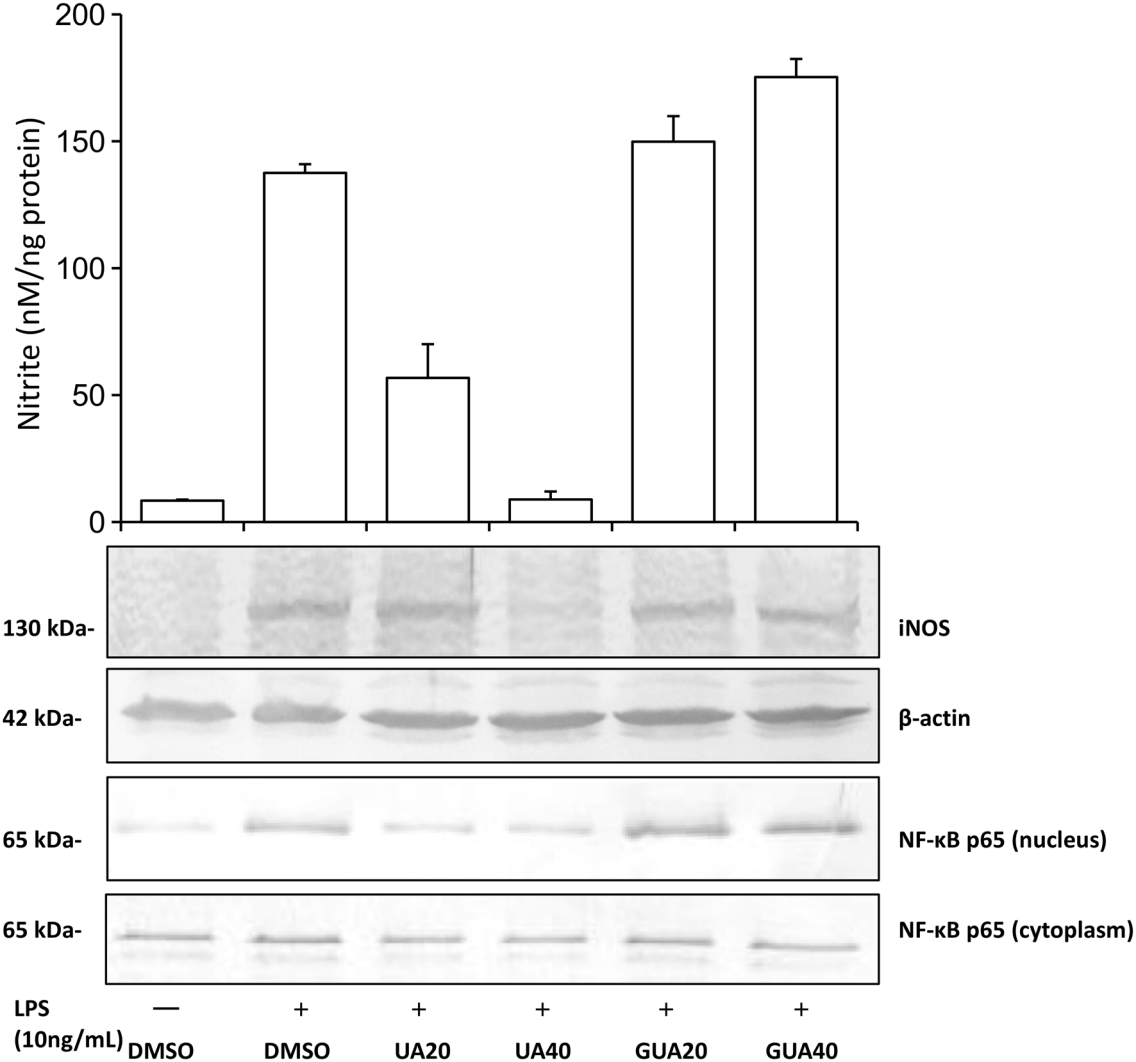
Effects of UA and GUA at the concentration of 40 and 20 μM on LPS-induced NO production, iNOS expression and NF-κB p65 nuclear translocation in RAW 264.7 macrophages. Inhibition of nitric oxide production by UA was statistically significant (*p* < 0.001, determined by ANOVA followed by Student’s *t* tests for unpaired data) at all concentrations tested. The increase of nitric oxide synthesis between GUA and UA was statistically highly significant (*p* < 0.001). Data were expressed as mean ± SD. Data shown are representative for three independent experiments

In order to bridge the current research with our previous studies on RAW 264.7 murine macrophages [13] the comparative examination of UA and GUA was additionally conducted using this cell model. Incubation of stimulated RAW 264.7 macrophages with increasing amounts of UA was associated with a concentration-dependent reduction of the NO production. The extent of inhibition was 62.5 ± 10.3% for 20 µM UA, and 99.6 ± 2.4% for 40 µM UA. GUA did not inhibit inducible nitric oxide synthesis. UA, but not GUA, was shown to decrease iNOS protein expression in a concentration-dependent manner what was associated with significant reduction of p65 nuclear translocation (Fig. 6).

**Fig. 7:**
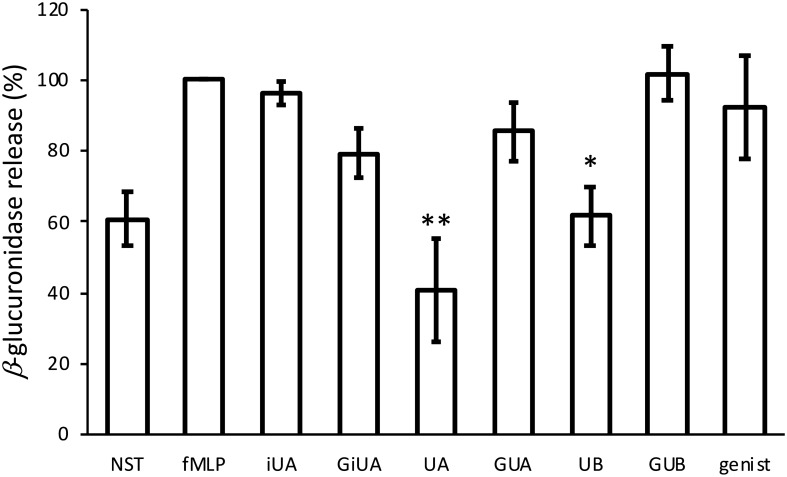
Effects of tested urolithins and respective glucuronides at the concentration of 40 μM on f-MLP-induced β-glucuronidase release from human primary neutrophhils. After isolation, neutrophils were resuspended in HBSS with iso-urolithin A, urolithin A and B (iUA, UA, UB) and their respective glucuronides (GiUA, GUA, GUB) at the concentration of 40 μM primed with cytochalasin B (10 μM) for 5 min and then stimulated with f-MLP (1 μM) for 10 min. Statistical significance: *p < 0.05, **p < 0.001 decrease versus stimulated control (Dunnettʼs post hoc test); fMLP—stimulated control; NST—non-stimulated control. Genist-genistein at the concentration of 40 μM (positive control). Data were expressed as mean ± SD of three separate experiments performed with cells isolated from independent donors. Mean ± SD and *p* values are provided in Supplementary material Table S7

Neutrophils are another type of immune cells contributing to the inflammatory response. They release granules containing various factors being cytotoxic for pathogenic microorganisms such as proteolytic enzymes and reactive oxygen species, which are responsible for tissue injury during persistent inflammation [28]. Studies conducted on human primary neutrophils isolated from peripheral venous blood have shown that UA and UB are able to inhibit the release of azurophilic granules (determined as the amount of excreted β-glucuronidase) (Fig. 7), while none of the tested compounds influenced the specific granule release which was monitored as the surface expression of CD66b (Fig. S4). The results were compared with the activity of positive control-genistein at the concentration of 40 μM, which is a broad specificity tyrosine kinase inhibitor known to inhibit neutrophil degranulation [29].

A key component of resolution of inflammation is ensuring that recruited neutrophils are cleared from the site of injury. Such clearance mechanisms prevent the inappropriate activation and the release of neutrophil microbial defense mechanisms and consequently host tissue injury. The processes leading to the removal of neutrophils include apoptosis and local efferocytosis by macrophages [28]. The studies on apoptosis have shown no cytotoxic activity on human primary neutrophils for any of the compounds and none of them was able to induce neutrophil apoptosis, neither in LPS-stimulated nor in non-stimulated cells. Roscovitine-CDK inhibitor at the concentration of 40 μM was used as a positive control (Fig. S2).

## Discussion

In the present study, the comparative examinations of in vitro anti-inflammatory activities of urolithins and their phase II metabolites were conducted on human immune cells. The examinations were performed for the most abundant urolithins characteristic of metabotypes A and B [7]: iUA, UA and UB as well as their respective glucuronide conjugates. Since recent data suggest that phenotype-based classification of macrophages in vivo is an oversimplification as phenotypes are very specific to their microenvironment [30], the comparative studies of compound’s activity were conducted on broad spectrum of macrophages origin: THP-1 human cell line-derived macrophages, RAW 264.7 murine macrophages and human primary macrophages. It was clearly confirmed that UA is the most active metabolite not only in terms of the inhibition of human immune cells’ inflammatory response induced by TLR4 receptor stimulation, but also towards stimulation of production of cytokines responsible for resolution of inflammation-IL-10 and TGF-β1. These results are congruent with previous studies conducted on THP-1 macrophages for UA, UB and urolithin C showing the strongest inhibition of UA on TNF-α production [12] as well as inhibition of TNF-α-induced MMP-9 production [31]. The mechanism of anti-inflammatory activity of UA and UB based on inhibition of NFκB translocation to the nucleus and involvement of MAPK phosphorylation in this process was for the first time determined by Gonzalez-Sarrias et al*.* on colon fibroblasts stimulated with IL-1β [16]. The same compounds were also tested on murine RAW 264.7 macrophages indicating exceptional activity of UA towards their inflammatory response, which was also demonstrated to depend on inhibition of NFκB p65 nuclear translocation [13]. The inhibition of NFκB pathway by UA was later confirmed on various murine cell models-macrophages and microglial cells. UA inhibited LPS-triggered production of NO, ROS and pro-inflammatory proteins in murine J774.1 macrophages, what depended on elevated autophagic flux, which in turn blocked NFκB p65 translocation to the nucleus. No influence on IκBα degradation was observed; thus the point of action of UA was located between the release from IκB and the nuclear translocation of p65 [32]. Further studies on LPS-stimulated RAW 264.7 macrophages indicated UA to inhibit pro-inflammatory mediators’ production by suppressing NOX-derived reactive oxygen species-mediated PI3-K/Akt/NF-κB and JNK/AP-1 and p38 signaling pathways. On this cell model UA was shown to inhibit degradation of IκBα; however, no influence on ERK1/2 phosphorylation was observed [10]. Studies on BV2 microglia cells confirmed UA to inhibit pro-inflammatory cytokines (TNF-α and IL-6) what was caused by the inhibition of p65 NFκB phosphorylation and acetylation [33]. UB also inhibited the production of NO and pro-inflammatory cytokines (TNF-α, IL-6 and IL-1β) and increased anti-inflammatory IL-10 in LPS-stimulated BV2 cells. UB suppressed NF-κB activity by inhibiting the phosphorylation and degradation of IκBα and the suppression of AP-1 activity in LPS-stimulated BV2 cells. In addition, UB suppressed the phosphorylation of JNK, ERK, and Akt, and enhanced the phosphorylation of AMPK. It was inactive in terms of p38 phosphorylation [34]. Nitrite analysis and qRT-PCR suggested that UA and UB reduced NO levels and suppressed mRNA levels of pro-inflammatory genes of TNF-α, IL-6, IL-1β, iNOS and COX-2 in LPS-treated BV2 microglia. Western blot revealed that tested urolithins decreased phosphorylation levels of Erk1/2, p38 MAPK and Akt, prevented the IκB-α phosphorylation and degradation and inhibited NF-κB p65 subunit phosphorylation and its nuclear translocation [15]. The anti-inflammatory activity of UA was also shown on different murine in vivo models. Anti-inflammatory properties of a pomegranate extract and its metabolite urolithin-A were demonstrated in a colitis rat model [11]. In vivo anti-inflammatory and antioxidant properties of ellagitannin metabolite urolithin A were also demonstrated on carrageenan-induced paw edema model in mice [8]. Orally applied UA was shown to inhibit p65 NFκB and p38 phosphorylation on in vivo neuroinflammation mouse model [35]. Treatment with UA attenuated colitis in animal models by restoring barrier dysfunction in addition to anti-inflammatory activities [36]. The anti-inflammatory activity of UA was previously shown on human chondrocytes. UA was inhibiting IL-1β-induced expression of iNOS, COX-2, TNF-α and IL-6, what depended on attenuated p65 nuclear translocation [37].

The hereby determined influence of UA on the inflammatory response of human macrophages (primary and cell line) unambiguously confirms previously established effects on murine in vitro and in vivo models. However, some discrepancies regarding the mechanism of action have to be addressed. Similarly, as in the previously published research, UA was indeed shown to attenuate the NF-κB p65 nuclear translocation; however, the effect was not exceptional in the comparison to its isomer iUA and was even weaker than for its glucuronide conjugates, which had no effect on TNF-α production. As in studies conducted by Boakye et al. although inhibition of NF-κB p65 translocation was observed, the degradation of IκBα was not prevented by any of the compounds. In contrast to earlier studies, no inhibition of SAPK/JNK and p38 phosphorylation was observed, while very strong induction of ERK1/2 phosphorylation for UA was determined. The induction of ERK1/2 phosphorylation was previously reported to serve as an anti-inflammatory signal that suppresses expression of NFκB-dependent inflammatory genes by inhibiting IKK activity. The increase in the level of pERK1/2 can exert the anti-inflammatory response and can contribute to the observed inhibition of TNF-α production [38]. The observed inconsistency in the mechanism of action of UA could be due to the fact that so far almost only murine in vitro and in vivo models were applied for its anti-inflammatory activity studies. It is known that the effects on signaling pathways in humans differ from those in mice when similar or even identical mutations are compared within the proteins involved. Because NF-κB and MAPKs pathways are involved in the infection control and the hygienic conditions as well as pathogens impinging on the lives of primates and rodents are different, the evolution adapted their functions according to the precise requirements of the two orders [39]. What is more, the data regarding the metabolism of ellagitannins to urolithins in rodents are showing either low urolithin production yield in the comparison to other mammals or domination of other type of metabolites, namely nasutins [24, 40–43].

Previous comparative study of UA and GUA regarding impact on the inflammatory response was conducted on human aortic endothelial cells stimulated with TNF-α. It has shown the decrease in the IL-8 production only for UA, PAI levels and monocyte adhesion attenuation only for GUA, while both compounds decreased the levels of CCL-2 [9]. Recent studies on human primary leukocytes have shown that UA and iUA are potent to reduce the formation of the 5-LOX/COX-2 pathway products (HKE_2_ and HKD_2_) through the attenuation of COX-2 and PGE2, while urolithin C was shown to reduce 5-HETE and LTB4 via inhibition of 5-LOX. The respective glucuronide and sulfate conjugates of UA and iUA were inactive [44]. In the present study no activity towards the production of pro- and anti-inflammatory cytokines was observed for phase II conjugates of urolithins. Despite significant inhibition of NFκB p65 translocation was unambiguously determined for GiUA, GUA and GUB, its consequences were not seen at the tested cytokine mRNA expression and protein level. Thus, we hypothesize, that in THP-1 macrophages the stimulation of ERK1/2 phosphorylation, which was determined for UA could play a role in its exceptional activity towards inhibition of TNF-α production. The loss of activity of urolithins due to phase II metabolism is also consistent with previous studies conducted on breast cancer and colon cancer cell lines. Glucuronidation was shown to decrease the anti-proliferative effects of UA and UB on colon cancer cell lines. Phase-II metabolism in HT-29 cells was a mechanism of cancer resistance against urolithins due to their conversion to glucuronide conjugates that exerted lower antiproliferative activity [23]. iUA, UA and UB exerted antiproliferative and antiestrogenic activities, but both their glucuronide and sulfate conjugates expressed much lower or no activities. In addition, the tested aglycones underwent phase II metabolism in breast cancer cell lines [22].

Urolithins are claimed to be actively glucuronidated in the large intestinal enterocytes before entering the bloodstream as their phase II metabolites. In consequence, the major metabolites present in the bloodstream and tissues are mainly urolithin glucuronide conjugates, whose maximal concentrations in the human plasma reach up to 35 μM for GUA, 0.745 μM for GiUA, and 7.3 μM for GUB. Significantly higher concentrations of the conjugates were detected in urine after the repeated consumption of ellagitannin-containing food products: 927–5330 μM for GUA and 550–6185 μM for GUB [7]. Taking into consideration the strong inhibitory activity of urolithins [in particular UA (which has been so far unambiguously confirmed on murine and human cell lines)], towards the inflammatory response of immune cells, the development of accelerated detoxifying mechanisms for these xenobiotics already in the gut wall is justified. Especially if the fact of urolithins production from commonly consumed food products is considered, what without sufficient phase II metabolism could negatively affect immune cells homeostasis in the healthy organism. As shown in current studies, the glucuronidation process is fully effective, as it leads to loss of impact on the inflammatory response of human immune cells.

The anti-inflammatory activity of urolithin aglycones applied at the concentration of 40 μM can be relevant to their potent interaction with the intestinal epithelium, as the levels of urolithins in feces were shown to reach up to 37–7180 μg/g of UA, 36–207 μg/g of iUA μM and 13–1894 μg/g of UB [7]. However, due to phase II metabolism already taking place in enterocytes, following absorption they are present in serum mainly as glucuronide conjugates GUA, GiUA, GUB at the concentrations, which in conducted study were unequivocally shown to be inactive. Despite applied concentration of urolithin aglycones in conducted in vitro studies is beyond the levels detected in serum following ingestion of ellagitannin-containing products, it needs to be noted that tissue distribution and metabolic disposition havenot been yet fully established, especially in the case of tissues with ongoing inflammation. In the previous studies we have shown that urolithin glucuronides are cleaved by human *β*-glucuronidase, which is released by human neutrophils from azurophilic granules upon stimulation of formyl peptide receptor-1 [24]. Based on the obtained results we hypothesized that the selective activation of urolithin glucuronides by β-glucuronidase present at high concentrations at the inflammation site could only locally increase the concentration of biologically active urolithin aglycones. Taking into consideration very low solubility of urolithins in aqueous environment, this process can lead to their precipitation in inflamed tissues resulting in significantly elevated concentrations of free aglycones at the inflammation site. Our hypothesis of on-site activation of urolithin glucuronides was partially confirmed in further in vivo studies conducted on rats with LPS-induced systemic inflammation [45].

## Conclusions

For the first time, comparative studies of the most abundant urolithins and their phase II conjugates were conducted on human immune cells. UA was unambiguously confirmed to be the most active metabolite in terms of inhibition of the inflammatory response both in THP-1 cell line as well as in primary macrophages. It was not only shown to attenuate NFκB p65 nuclear translocation, what is fully congruent with previous studies, but was also to significantly stimulate ERK1/2 phosphorylation, what stands in contradiction with previous in vitro and in vivo examinations conducted on murine models. The determined moderate discrepancies in the mechanism of action could be the consequence of the differences in mechanisms of immune response between murine and human cells, what should be considered while designing further in vitro and in vivo studies. Phase II metabolism of urolithins was clearly shown to cause their deactivation. Urolithin glucuronides were not active towards any of the tested signaling protein expression or production; however, they were clearly shown to inhibit NFκB p65 nuclear translocation. In order to elucidate what are the consequences of this attenuation at the cytokine and chemokine level, further studies including their broader array are necessary.

## Electronic supplementary material

Below is the link to the electronic supplementary material.

Supplementary file1 (PDF 78 kb)

Supplementary file2 (PDF 351 kb)

## Abbreviations

iUA: Iso-urolithin A
UA: Urolithin A
UB: Urolithin B
Genist: Genistein
GiUA: Iso-urolithin A 3-*O*-glucuronide
GUA: Urolithin A 3-*O*-glucuronide and 8-*O*-glucuronide
GUB: Urolithin B 3-*O*-glucuronide
LPS: Lipopolisachcharide
Parth: Parthenolide
PMA: Phorbol myristate acetate
