# Supplementary material for: Comparative studies of urolithins and their phase II metabolites on macrophage and neutrophil functions
Source: Eur J Nutr. 2020 Sep 22;60(4):1957–72. doi: 10.1007/s00394-020-02386-y (PMC8137622; doi:10.1007/s00394-020-02386-y)
Supplement: Supplementary file 1 — Supplementary file1 (PDF 78 kb) [file 394_2020_2386_MOESM1_ESM.pdf]

Supplementary material Tables S1-S7

European Journal of Nutrition

Comparative studies of urolithins and their phase II metabolites on macrophage and neutrophil functions.

Aneta Bobowska[1], Sebastian Granica[1], Agnieszka Filipek[1], Matthias F. Melzig[2], Thomas Moeslinger[3], Jürgen Zentek[4], Aleksandra Kruk[1], Jakub P. Piwowarski[1,2,4]

[1]Department of Pharmacognosy and Molecular Basis of Phytotherapy, Faculty of Pharmacy, Medical University of Warsaw, Warsaw, Poland

[2]Department of Pharmaceutical Biology, Institute of Pharmacy, Freie Universität Berlin, Berlin, Germany

[3]Institute of Physiology, Center for Physiology and Pharmacology, Medical University of Vienna, Vienna, Austria

[4]Institute of Animal Nutrition, Freie Universität Berlin, Berlin, Germany

Corresponding author:

dr hab. Jakub Patryk Piwowarski, Department of Pharmacognosy and Molecular Basis of Phytotherapy, Faculty of Pharmacy, Medical University of Warsaw, Banacha 1, 02-097 Warsaw, Poland. e-mail: jakub.piwowarski@wum.edu.pl

ORCID: http://orcid.org/0000-0002-5011-0983

Table S1. Effects of tested urolithins and respective glucuronides at the concentration of 40 µM on viability of THP-1 macrophages. PMA-differentiated THP-1 cells were preincubated for 1h with iso-urolithin A, urolithin A and B (iUA, UA, UB), their respective glucuronides (GiUA, GUA, GUB) at the concentration of 40 µM, parthenolide (Parth) at the concentration of 5 µM and stimulated with LPS (10 ng/mL) for 24 h. MTT test was performed as described in Materials and methods section.

			post hoc (Dunnett)		*post hoc* (Tukey)								
	**viability (%)**	**SD**	***p* (<LPS)**	***p* (>LPS)**		**NST**	**LPS**	**iUA**	**GiUA**	**UA**	**GUA**	**UB**	**GUB**
**NST**	109,4	3,9	0,999954	0,058926	**NST**								
**LPS**	100,0	2,1			**LPS**	0,380802							
**iUA**	103,3	11,7	0,986367	0,554204	**iUA**	0,855963	0,993753						
**GiUA**	101,8	6,5	0,959475	0,726273	**GiUA**	0,655934	0,999920	0,999982					
**UA**	94,8	4,7	0,325675	0,997400	**UA**	0,026668	0,903563	0,406000	0,655506				
**GUA**	94,6	5,0	0,300016	0,997917	**GUA**	0,022926	0,879444	0,368967	0,614636	1,000000			
**UB**	106,2	11,6	0,998179	0,285092	**UB**	0,998662	0,912834	0,999304	0,988082	0,282639	0,258121		
**GUB**	99,9	4,4	0,873140	0,886020	**GUB**	0,366829	1,000000	0,992357	0,999879	0,912758	0,890002	0,905373	
**Parthenolide**	99,7	3,3	0,862237	0,895542	**Parthenolide**	0,343553	1,000000	0,994194	0,999883	0,955880	0,942433	0,891711	1,000000

Table S2. Effects of tested urolithins and respective glucuronides at the concentration of 40 μM on apoptosis of non-stimulated (A) and LPS-stimulated (100 ng/ml) (B) human primary neutrophils. Neutrophils were incubated with iso-urolithin A, urolithin A and B (iUA, UA, UB), their respective glucuronides (GiUA, GUA, GUB) at the concentration of 40 μM, roscovitine at the concentration of 40 μM and/or stimulated with LPS (10 ng/mL) for 20 h. Neutrophils' viability and apoptosis was determined by staining with propidium iodide (PI) and Annexin V-FITC after 6h in non-stimulated cells (A) and after 20h in LPS-stimulated cells.

**without LPS (6h)**			*post hoc* (Dunnett)				*post hoc* (Dunnett)				*post hoc* (Dunnett)	
	**viable (%)**	**SD**	***p* (<NST)**	***p* (>NST)**	**early apoptosis (%)**	**SD**	***p* (<NST)**	***p* (>NST)**	**late apoptosis (%)**	**SD**	***p* (<NST)**	***p* (>NST)**
**NST**	88,8	4,4			9,1	2,9			1,9	1,8		
**LPS**	93,2	3,5	0,999945	0,077183	5,2	2,7	0,035004	0,999969	1,4	0,9	0,572742	0,988726
**iUA**	90,4	2,4	0,986898	0,595769	8,4	2,2	0,730938	0,968313	1,1	0,5	0,397543	0,996664
**GiUA**	90,5	2,0	0,990130	0,999999	8,7	1,9	0,813820	0,943284	0,7	0,2	0,162098	0,999821
**UA**	91,2	2,2	0,996629	0,398937	6,9	1,2	0,274710	0,998804	1,7	0,9	0,784393	0,954204
**GUA**	89,9	2,5	0,972962	0,707114	9,0	1,6	0,869998	0,911451	1,0	0,6	0,308857	0,998373
**UB**	93,1	1,1	0,999939	0,083205	5,8	0,9	0,074388	0,999947	0,9	0,4	0,291258	0,998608
**GUB**	90,7	2,6	0,992621	0,508888	8,2	2,1	0,675748	0,977990	0,9	0,4	0,279834	0,998746
**Roscovitine**	29,3	0,6	0,000019	0,999978	67,4	0,4	0,999978	0,000019	3,1	0,3	0,999086	0,247267

**with LPS 20h**			*post hoc* (Dunnett)				*post hoc* (Dunnett)				*post hoc* (Dunnett)	
	**viable (%)**	**SD**	***p* (<LPS)**	***p* (>LPS)**	**early apoptosis (%)**	**SD**	***p* (<LPS)**	***p* (>LPS)**	**late apoptosis (%)**	**SD**	***p* (<LPS)**	***p* (>LPS)**
**NST**	34,8	3,8	0,000005	0,999978	43,7	10,0	0,999979	0,000026	20,0	5,1	0,999978	0,000005
**LPS**	82,6	6,1			13,6	5,0			3,2	0,9		
**iUA**	79,1	5,9	0,637201	0,970780	17,2	5,2	0,985812	0,530331	2,8	1,1	0,820630	0,908687
**GiUA**	76,1	10,4	0,385180	0,994085	15,6	4,8	0,959505	0,703248	5,4	3,4	0,990567	0,453445
**UA**	78,5	5,6	0,585185	0,978580	17,6	4,3	0,988943	0,490735	3,4	1,1	0,889707	0,847534
**GUA**	76,5	9,7	0,417738	0,992573	16,6	5,1	0,978028	0,601980	5,1	3,2	0,984787	0,528806
**UB**	86,6	4,7	0,976311	0,602008	9,2	3,3	0,456219	0,991159	3,1	1,7	0,852507	0,885643
**GUB**	75,5	10,5	0,378365	0,994367	16,2	6,3	0,968035	0,664256	6,8	3,8	0,998470	0,224425
**Roscovitine**	3,8	2,9	0,000005	0,999978	82,7	0,8	0,999979	0,000024	11,7	1,1	0,999978	0,001460

Table S3. Effects of tested urolithins and respective glucuronides at the concentration of 40 µM on LPS-induced TNF-α and IL-10 production. PMA-differentiated THP-1 cells were preincubated for 1h with iso-urolithin A, urolithin A and B (iUA, UA, UB) and their respective glucuronides (GiUA, GUA, GUB) at the concentration of 40 µM and stimulated with LPS (10 ng/mL).

			post hoc (Dunnett)		*post hoc* (Tukey)								
	**TNF-α 3h (%)**	**SD**	***p* (<LPS)**	***p* (>LPS)**		**NST**	**LPS**	**iUA**	**GiUA**	**UA**	**GUA**	**UB**	**GUB**
**NST**	0,0	0,0	0,000022	0,999963	**NST**								
**LPS**	100,0	4,0			**LPS**	0,000136							
**iUA**	93,1	11,7	0,108434	0,999962	**iUA**	0,000136	0,540174						
**GiUA**	106,3	8,7	0,999921	0,283703	**GiUA**	0,000136	0,908662	0,115742					
**UA**	55,7	8,1	0,000022	0,999963	**UA**	0,000136	0,000136	0,000136	0,000136				
**GUA**	97,7	8,4	0,751957	0,994840	**GUA**	0,000136	0,999597	0,959170	0,638124	0,000136			
**UB**	89,1	8,9	0,004432	0,999963	**UB**	0,000136	0,054933	0,964476	0,011138	0,000136	0,423909		
**GUB**	98,1	9,6	0,791211	0,992500	**GUB**	0,000136	0,999842	0,905742	0,696014	0,000136	1,000000	0,272747	
**Parthenolide**	33,2	5,5	0,000022	0,999963	**Parthenolide**	0,000136	0,000136	0,000136	0,000136	0,000166	0,000136	0,000136	0,000136

			post hoc (Dunnett)		*post hoc* (Tukey)								
	**TNF-α 6h (%)**	**SD**	***p* (<LPS)**	***p* (>LPS)**		**NST**	**LPS**	**iUA**	**GiUA**	**UA**	**GUA**	**UB**	**GUB**
**NST**	2,9	1,0	0,000021	0,999967	**NST**								
**LPS**	100,0	12,5			**LPS**	0,000144							
**iUA**	134,6	9,8	0,999967	0,000021	**iUA**	0,000144	0,000144						
**GiUA**	109,5	9,9	0,999959	0,139040	**GiUA**	0,000144	0,717004	0,000777					
**UA**	75,2	7,7	0,000028	0,999967	**UA**	0,000144	0,000924	0,000144	0,000145				
**GUA**	94,1	8,7	0,438330	0,999323	**GUA**	0,000144	0,973293	0,000144	0,127700	0,024815			
**UB**	112,1	12,3	0,999967	0,041287	**UB**	0,000144	0,398475	0,003483	0,999905	0,000144	0,038329		
**GUB**	101,5	10,1	0,973598	0,862002	**GUB**	0,000144	0,999999	0,000146	0,863149	0,000440	0,906987	0,574407	
**Parthenolide**	41,1	8,8	0,000021	0,999967	**Parthenolide**	0,000144	0,000144	0,000144	0,000144	0,000145	0,000144	0,000144	0,000144

			post hoc (Dunnett)		*post hoc* (Tukey)								
	**IL-10 24h (%)**	**SD**	***p* (<LPS)**	***p* (>LPS)**		**NST**	**LPS**	**iUA**	**GiUA**	**UA**	**GUA**	**UB**	**GUB**
**NST**	74,2	21,6	0,000096	0,999962	**NST**								
**LPS**	100,0	5,0			**LPS**	0,001091							
**iUA**	141,4	21,6	0,999962	0,000023	**iUA**	0,000134	0,000466						
**GiUA**	98,3	15,7	0,903330	0,975355	**GiUA**	0,154255	1,000000	0,000286					
**UA**	97,5	24,3	0,869079	0,984106	**UA**	0,081641	0,999997	0,000242	1,000000				
**GUA**	110,4	7,4	0,999863	0,358578	**GUA**	0,001233	0,939385	0,019344	0,902516	0,821682			
**UB**	115,7	15,9	0,999962	0,081222	**UB**	0,000159	0,534456	0,098237	0,561303	0,332117	0,999316		
**GUB**	111,2	11,1	0,999939	0,305872	**GUB**	0,000902	0,909641	0,025232	0,865956	0,768436	1,000000	0,999796	
**Parthenolide**	69,4	2,8	0,015201	0,999962	**Parthenolide**	0,999992	0,369049	0,000157	0,450495	0,486247	0,072814	0,024938	0,062485

Table S4. Effects of tested urolithins and respective glucuronides at the concentration of 40 μM on LPS-induced TNF-α and TGF-β1 mRNA expression in THP-1 macrophages. PMA-differentiated THP-1 cells were preincubated for 1h with iso-urolithin A, urolithin A and B (iUA, UA, UB) and their respective glucuronides (GiUA, GUA, GUB) at the concentration of 40 μM and stimulated with LPS (10 ng/mL) for 24 h. Real-time RT-PCR analysis was performed as described in Materials and methods section. Changes in mRNA expression were normalized to β-actin. Parthenolide at the concentration of 5 μM was used as a positive control.

			post hoc (Dunnett)		*post hoc* (Tukey)								
	**TNF-α mRNA expression**	**SD**	***p* (<LPS)**	***p* (>LPS)**		**NST**	**LPS**	**iUA**	**GiUA**	**UA**	**GUA**	**UB**	**GUB**
**NST**	0,307	0,100	0,000021	0,999977	**NST**								
**LPS**	1,000	0,000			**LPS**	0,000157							
**iUA**	1,656	0,357	0,999977	0,000023	**iUA**	0,000151	0,000167						
**GiUA**	1,259	0,169	0,999963	0,048627	**GiUA**	0,000151	0,264518	0,015475					
**UA**	0,696	0,050	0,019004	0,999974	**UA**	0,018594	0,118597	0,000151	0,000376				
**GUA**	1,075	0,099	0,982345	0,628681	**GUA**	0,000152	0,997784	0,000290	0,687112	0,023592			
**UB**	0,885	0,037	0,446890	0,994711	**UB**	0,000303	0,965475	0,000152	0,026219	0,659436	0,647125		
**GUB**	0,966	0,069	0,793004	0,947672	**GUB**	0,000166	0,999994	0,000157	0,145385	0,221474	0,974735	0,996228	
**Parthenolide**	0,573	0,070	0,000996	0,999977	**Parthenolide**	0,237884	0,007535	0,000151	0,000158	0,948326	0,001252	0,100151	0,016858

			post hoc (Dunnett)		*post hoc* (Tukey)								
	**TGF-β1 mRNA expression**	**SD**	***p* (<LPS)**	***p* (>LPS)**		**NST**	**LPS**	**iUA**	**GiUA**	**UA**	**GUA**	**UB**	**GUB**
**NST**	1,938	0,319	0,999977	0,000020	**NST**								
**LPS**	1,000	0,000			**LPS**	0,000152							
**iUA**	1,276	0,061	0,999942	0,077760	**iUA**	0,000366	0,382545						
**GiUA**	1,024	0,129	0,927998	0,836339	**GiUA**	0,000152	1,000000	0,498991					
**UA**	1,603	0,250	0,999977	0,000125	**UA**	0,167581	0,000972	0,187941	0,001544				
**GUA**	1,029	0,109	0,935048	0,822715	**GUA**	0,000152	1,000000	0,526359	1,000000	0,001722			
**UB**	1,350	0,167	0,999973	0,021201	**UB**	0,001312	0,130620	0,999332	0,190211	0,494948	0,206003		
**GUB**	0,980	0,053	0,846782	0,921913	**GUB**	0,000152	1,000000	0,298611	0,999989	0,000683	0,999973	0,094077	
**Parthenolide**	1,256	0,184	0,999905	0,105615	**Parthenolide**	0,000289	0,478509	1,000000	0,602079	0,137741	0,629843	0,996436	0,383650

Table S5. Effects of tested urolithins and respective glucuronides at the concentration of 40 µM on LPS-induced TNF-α. PBMCs were incubated with iso-urolithin A, urolithin A and B (iUA, UA, UB) and their respective glucuronides (GiUA, GUA, GUB) at the concentration of 40 µM and stimulated with LPS (100 ng/mL).

			post hoc (Dunnett)		*post hoc* (Tukey)							
	**TNF-α (%)**	**SD**	***p* (<LPS)**	***p* (>LPS)**		**NST**	**LPS**	**iUA**	**GiUA**	**UA**	**GUA**	**UB**
**NST**	0,528	0,587	0,000024	0,999979	**NST**							
**LPS**	100,000	0,000			**LPS**	0,000150						
**iUA**	45,587	3,325	0,000029	0,999979	**iUA**	0,002912	0,000407					
**GiUA**	95,550	5,463	0,767267	0,982755	**GiUA**	0,000150	0,999774	0,000963				
**UA**	8,198	3,478	0,000024	0,999979	**UA**	0,869576	0,000150	0,017525	0,000150			
**GUA**	131,429	24,683	0,999979	0,000526	**GUA**	0,000150	0,003358	0,000150	0,024751	0,000150		
**UB**	95,059	10,860	0,709656	0,988972	**UB**	0,000150	0,998317	0,001069	1,000000	0,000150	0,003282	
**GUB**	101,317	4,807	0,951260	0,883040	**GUB**	0,000150	1,000000	0,000332	0,998762	0,000150	0,019703	0,992691

Table S6. Effects of tested urolithins and respective glucuronides at the concentration of 40 μM on LPS-induced NF-κBp65 nuclear translocation and ERK1/2 phosphorylation in THP-1 macrophages. PMA-differentiated THP-1 cells were preincubated for 1h with iso-urolithin A, urolithin A and B (iUA, UA, UB) and their respective glucuronides (GiUA, GUA, GUB) at the concentration of 40 μM and stimulated with LPS (10 ng/mL) for 35 min. Parthenolide at the concentration of 5 μM was used as a positive control. Western blotting analysis was performed as described in Materials and methods. Densitometric analysis was performed using ImageJ software. Changes in p65 protein levels in nuclear fractions were normalized to p65 protein levels in cytosolic fractions. The changes in phospho-ERK1/2 were normalized to total ERK1/2.

			post hoc (Dunnett)		*post hoc* (Tukey)								
	**p65 nuclear translocation**	**SD**	***p* (<LPS)**	***p* (>LPS)**		**NST**	**LPS**	**iUA**	**GiUA**	**UA**	**GUA**	**UB**	**GUB**
**NST**	0,000	0,000	0,000020	0,999971	**NST**								
**LPS**	1,000	0,000			**LPS**	0,000143							
**iUA**	0,683	0,020	0,006501	0,999971	**iUA**	0,000143	0,046530						
**GiUA**	0,514	0,112	0,000049	0,999971	**GiUA**	0,000232	0,000387	0,715946					
**UA**	0,782	0,032	0,045704	0,999923	**UA**	0,000143	0,186041	0,981363	0,153234				
**GUA**	0,405	0,117	0,000020	0,999971	**GUA**	0,003801	0,000146	0,120306	0,964913	0,008669			
**UB**	0,831	0,042	0,192656	0,999520	**UB**	0,000143	0,712987	0,832413	0,047095	0,999862	0,001964		
**GUB**	0,483	0,112	0,000029	0,999971	**GUB**	0,000418	0,000223	0,501859	0,999995	0,073272	0,996111	0,019834	
**Parthenolide**	0,000	0,000	0,000020	0,999971	**Parthenolide**	1,000000	0,000143	0,000143	0,000232	0,000143	0,003801	0,000143	0,000418

			post hoc (Dunnett)		*post hoc* (Tukey)								
	**ERK1/2 phosphorylation**	**SD**	***p* (<LPS)**	***p* (>LPS)**		**NST**	**LPS**	**iUA**	**GiUA**	**UA**	**GUA**	**UB**	**GUB**
**NST**	0,170	0,094	0,000082	0,999553	**NST**								
**LPS**	1,000	0,000			**LPS**	0,661334							
**iUA**	1,921	0,423	0,999869	0,049058	**iUA**	0,015567	0,043506						
**GiUA**	1,379	0,260	0,987035	0,580605	**GiUA**	0,205182	0,994464	0,950585					
**UA**	3,153	0,777	0,999977	0,000226	**UA**	0,000160	0,001774	0,187516	0,013816				
**GUA**	1,140	0,214	0,944748	0,800609	**GUA**	0,468306	0,999997	0,726788	0,999793	0,003760			
**UB**	1,042	0,077	0,908841	0,865942	**UB**	0,603145	1,000000	0,593587	0,997531	0,002220	1,000000		
**GUB**	0,791	0,064	0,744861	0,962440	**GUB**	0,898517	0,999927	0,278225	0,923218	0,000623	0,996912	0,999712	
**Parthenolide**	0,793	0,092	0,746568	0,962007	**Parthenolide**	0,896919	0,999932	0,280234	0,924549	0,000629	0,997031	0,999729	1,000000

Table S7. Effects of tested urolithins and respective glucuronides at the concentration of 40 μM on f-MLP-induced β-glucuronidase release from human primary neutrophhils. After isolation, neutrophils were resuspended in HBSS with iso-urolithin A, urolithin A and B (iUA, UA, UB) and their respective glucuronides (GiUA, GUA, GUB) at the concentration of 40 μM primed with cytochalasin B (10 μM) for 5 min and then stimulated with f-MLP (1 μM) for 10 min. Genistein at the concentration of 40 μM was used as a positive control.

			post hoc (Dunnett)		*post hoc* (Tukey)								
	**β-glucuronidase (%)**	**SD**	***p* (<LPS)**	***p* (>LPS)**		**NST**	**LPS**	**iUA**	**GiUA**	**UA**	**GUA**	**UB**	**GUB**
**NST**	60,9	7,6	0,013047	0,999972	**NST**								
**LPS**	100,0	0,0			**LPS**	0,087996							
**iUA**	96,2	3,5	0,788193	0,926403	**iUA**	0,145624	0,999999						
**GiUA**	79,2	6,8	0,308796	0,996707	**GiUA**	0,721582	0,888322	0,961493					
**UA**	40,8	14,6	0,000917	0,999978	**UA**	0,994139	0,013853	0,006582	0,248087				
**GUA**	85,4	8,4	0,457707	0,990301	**GUA**	0,466331	0,984274	0,994364	0,999965	0,044542			
**UB**	61,8	8,3	0,030522	0,999958	**UB**	0,999640	0,253034	0,213364	0,956335	0,781710	0,665537		
**GUB**	101,6	7,6	0,896030	0,839290	**GUB**	0,070802	1,000000	0,999963	0,843751	0,002479	0,934993	0,097136	
**Genistein**	92,2	14,6	0,679385	0,962420	**Genistein**	0,233997	0,999809	0,999997	0,992350	0,013444	0,999779	0,349463	0,997926
