# Supplementary material for: Comparative studies of urolithins and their phase II metabolites on macrophage and neutrophil functions
Source: Eur J Nutr. 2020 Sep 22;60(4):1957–72. doi: 10.1007/s00394-020-02386-y (PMC8137622; doi:10.1007/s00394-020-02386-y)
Supplement: Supplementary file 2 — Supplementary file2 (PDF 351 kb) [file 394_2020_2386_MOESM2_ESM.pdf]

Supplementary material Figures S1-S4

European Journal of Nutrition

Comparative studies of urolithins and their phase II metabolites on macrophage and neutrophil functions.

Aneta Bobowska[1], Sebastian Granica[1], Agnieszka Filipek[1], Matthias F. Melzig[2], Thomas Moeslinger[3], Jürgen Zentek[4], Aleksandra Kruk[1], Jakub P. Piwowarski[1,2,4]

[1]Department of Pharmacognosy and Molecular Basis of Phytotherapy, Faculty of Pharmacy, Medical University of Warsaw, Warsaw, Poland

[2]Department of Pharmaceutical Biology, Institute of Pharmacy, Freie Universität Berlin, Berlin, Germany

[3]Institute of Physiology, Center for Physiology and Pharmacology, Medical University of Vienna, Vienna, Austria

[4]Institute of Animal Nutrition, Freie Universität Berlin, Berlin, Germany

Corresponding author:

dr hab. Jakub Patryk Piwowarski, Department of Pharmacognosy and Molecular Basis of Phytotherapy, Faculty of Pharmacy, Medical University of Warsaw, Banacha 1, 02-097 Warsaw, Poland. e-mail: jakub.piwowarski@wum.edu.pl

ORCID: http://orcid.org/0000-0002-5011-0983

Figure S1. Effects of tested urolithins and respective glucuronides at the concentration of 40 µM on viability of THP-1 macrophages. PMA-differentiated THP-1 cells were preincubated for 1h with iso-urolithin A, urolithin A and B (iUA, UA, UB), their respective glucuronides (GiUA, GUA, GUB) at the concentration of 40 µM, parthenolide (Parth) at the concentration of 5 µM and stimulated with LPS (10 ng/mL) for 24 h. MTT test was performed as described in Materials and methods section. *$p < 0.05$, **$p < 0.001$ versus stimulated control (Dunnett's *post hoc* test); LPS—stimulated control; NST—non-stimulated control. Three separate experiments were conducted in triplicate. Mean ± SD and p values are provided in supporting Information Table S1.

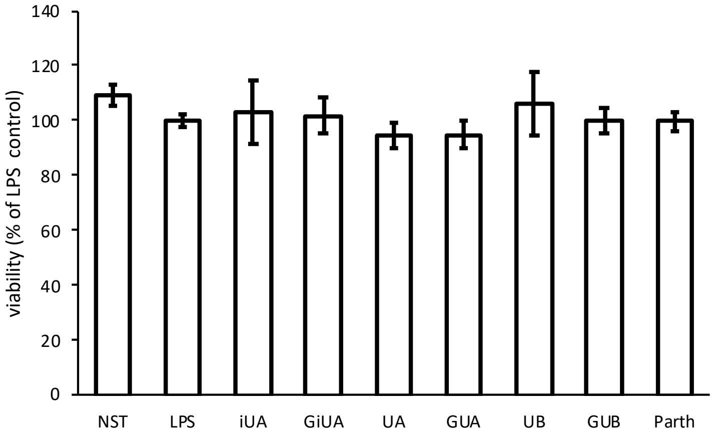

Figure S2. Effects of tested urolithins and respective glucuronides at the concentration of 40 µM on apoptosis of non-stimulated (A) and LPS-stimulated (100 ng/ml) (B) human primary neutrophils. Neutrophils were incubated with iso-urolithin A, urolithin A and B (iUA, UA, UB), their respective glucuronides (GiUA, GUA, GUB) at the concentration of 40 µM and/or stimulated with LPS (10 ng/mL) for 20 h. Neutrophils' viability and apoptosis was determined by staining with propidium iodide (PI) and Annexin V-FITC after 6h in non-stimulated cells (A) and after 20h in LPS-stimulated cells. Statistical significance: *$p < 0.05$, **$p < 0.001$ versus stimulated control (Dunnett's post hoc test); LPS—stimulated control; NST—non-stimulated control. Roscovitine (rosc) at the concentration of 40 µM was used as a positive control. Data were expressed as mean ± SD of four separate experiments performed with cells isolated from independent donors. Mean ± SD and *p* values are provided in supporting Information Table S2.

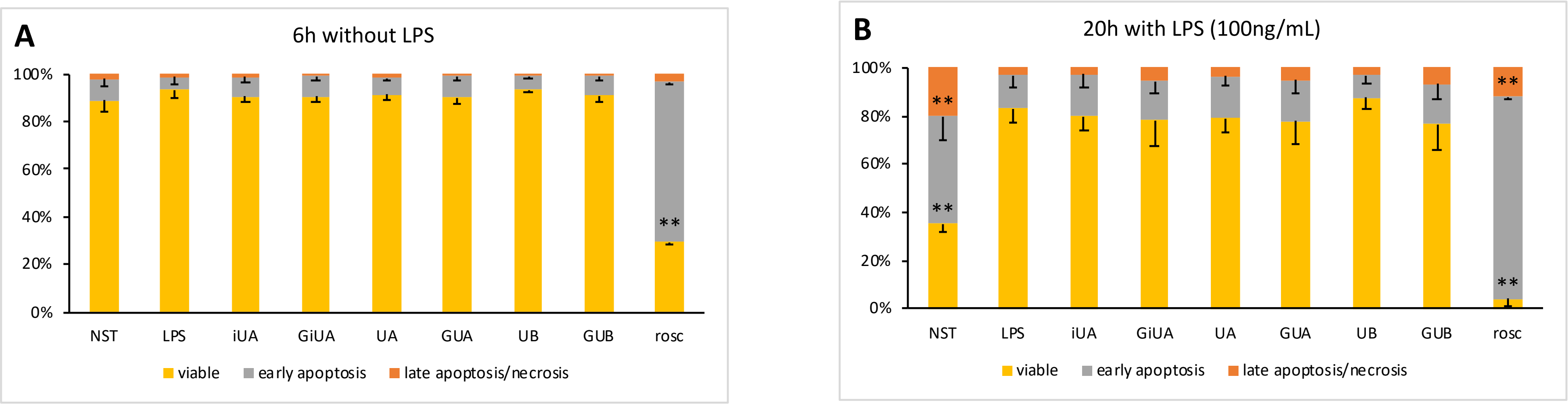

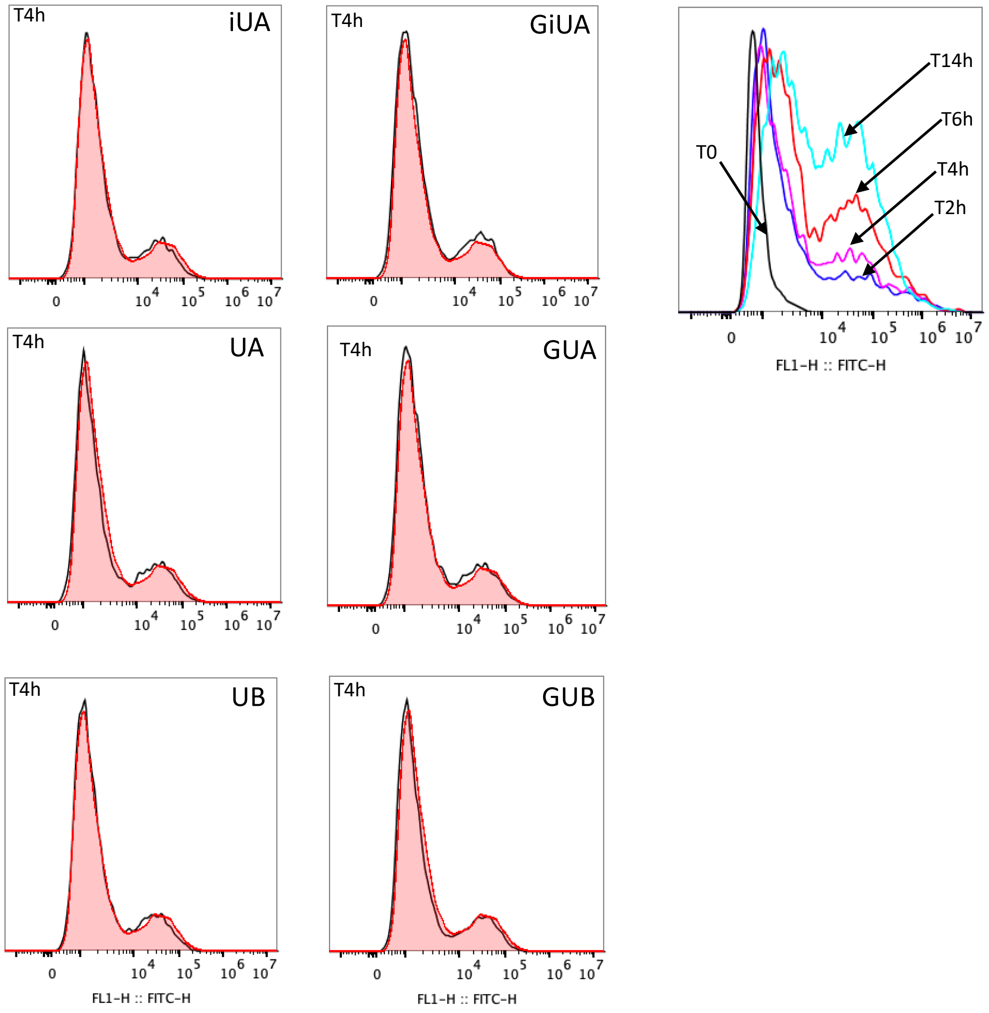

Figure S3. Effects of tested urolithins and respective glucuronides at the concentration of 40 µM on phagocytosis of IgG-FITC-coated beads by THP-1 macrophages. THP-1 macrophages were incubated with iso-urolithin A, urolithin A and B (iUA, UA, UB) and their respective glucuronides (GiUA, GUA, GUB) at the concentration of 40 µM for 1h. After treatment with compounds, 20 µL of FITC labelled beads suspension was added to the culture and the cells were incubated at 37 °C for 4h. Data are representative for three independent experiments.

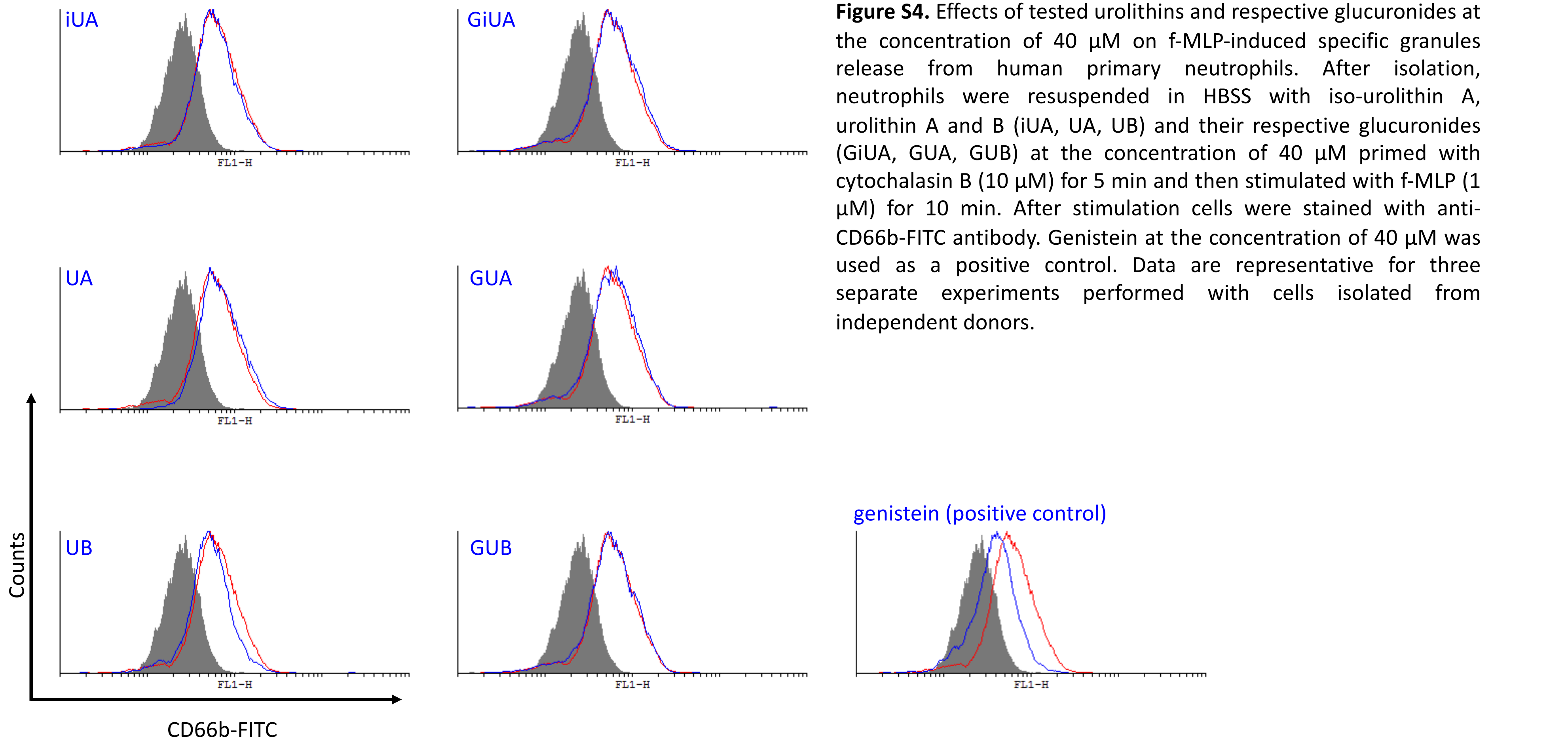

Figure S4. Effects of tested urolithins and respective glucuronides at the concentration of 40 µM on f-MLP-induced specific granules release from human primary neutrophils. After isolation, neutrophils were resuspended in HBSS with iso-urolithin A, urolithin A and B (iUA, UA, UB) and their respective glucuronides (GiUA, GUA, GUB) at the concentration of 40 µM primed with cytochalasin B (10 µM) for 5 min and then stimulated with f-MLP (1 µM) for 10 min. After stimulation cells were stained with anti-CD66b-FITC antibody. Genistein at the concentration of 40 µM was used as a positive control. Data are representative for three separate experiments performed with cells isolated from independent donors.
